# The prognostic role of PSMD14 in head and neck squamous cell carcinoma

**DOI:** 10.1007/s00432-022-04072-4

**Published:** 2022-06-25

**Authors:** Julia Schnoell, Alexandra Scheiflinger, Sega Al-Gboore, Lorenz Kadletz-Wanke, Lukas Kenner, Gregor Heiduschka, Bernhard J. Jank

**Affiliations:** 1 Department of Otorhinolaryngology, Head and Neck Surgery, Medical University of Vienna, Waehringer Guertel 18-20, 1090 Vienna, Austria; 2 Department of Pathology, Medical University of Vienna, Vienna, Austria; 3 Christian Doppler Laboratory for Applied Metabolomics, Vienna, Austria; 4 Unit of Laboratory Animal Pathology, University of Veterinary Medicine, Vienna, Austria; 5 CBmed GmbH-Center for Biomarker Research in Medicine, Graz, Styria Austria

**Keywords:** PSMD14, Biomarker, Head and neck, Cancer, Proteasome

## Abstract

**Purpose:**

PSMD14 is an essential protein for proteasomal degradation. Inhibition of this protein disrupts homeostasis and inhibits cancer cell viability. Overexpression of PSMD14 was associated with advanced cancer characteristics and a worse prognosis in various carcinomas. This study aimed to analyze PSMD14 copy number variation, mRNA and protein expression in HNSCC, and its role as an independent prognostic biomarker.

**Methods:**

PSMD14 mRNA expression and copy number variations were analyzed in “The Cancer Genome Atlas (TCGA)” in 510 patients. Protein expression was evaluated using immunohistochemistry in a second cohort including 115 patients. PSMD14 levels were analyzed for correlation with clinicopathological data, overall and disease-free survival.

**Results:**

PSMD14 mRNA expression and copy number variation were high in 44 and 50% of patients, respectively. Protein expression of PSMD14 was high in 56%. In both cohorts, high PSMD14 levels were associated with advanced staging. High PSMD14 mRNA expression was additionally associated with a worse prognosis in univariable analysis. However, after correction for possible confounders, PSMD14 mRNA was not an independent prognostic marker.

**Conclusion:**

PSMD14 is commonly expressed in HNSCC patients and associated with advanced stages. High expression of PSMD14 mRNA was associated with a worse outcome. However, this may be a result of the association of PSMD14 with poor prognosticators. Based on our study, further evaluation of PSMD14 as a prognostic marker and potential therapeutic target is warranted.

## Introduction

As the sixth most common cancer worldwide, head and neck squamous cell carcinoma (HNSCC) affects approximately 900,000 patients annually. The incidence of this disease is rising, while therapeutic options have only marginally improved (Johnson et al. 2020; Pulte and Brenner 2010). Currently, the only applied biomarker for risk stratification and to guide therapeutic decisions for oropharyngeal squamous cell carcinoma is the detection of HPV (Mirghani and Blanchard 2018; Tawk et al. 2022). Thus, there is an urgent need for novel biomarkers and new therapeutic options in HNSCC.

The 26S proteasome non-ATPase regulatory subunit 14 (PSMD14), also known as Rpn11 or POH1, is an essential component of the 26S proteasome. The proteasome is essential for protein degradation and is involved in protein homeostasis, cell division, and transcriptional regulation. Proteins are marked for proteasomal degradation by polyubiquitination. While ubiquitin is recognized by the 19S regulatory subunit, the 20s core subunit mediates proteolysis (Li et al. 2017). PSMD14 is located in the 19S regulatory subunit and cleaves ubiquitin directly prior to the proteins’ degradation (Shin et al. 2020). Without removal of ubiquitin, the protein is too bulky to enter the proteasome. Thus, inhibition of PSMD14 disrupts protein homeostasis and leads to apoptosis in cancer cells (Li et al. 2017; Jing et al. 2021a, b).

High PSMD14 mRNA and protein expression was associated with a worse prognosis in several cancers, including HNSCC (Jing et al. 2021a; Lv et al. 2019; Wang et al. 2019; Zhu et al. 2018; Luo et al. 2017; Song et al. 2017; Zhang et al. 2020; Lei et al. 2021). However, multivariable analysis was rarely performed, and thus the role as an independent prognostic marker is unclear. Since high expression of PSMD14 is commonly linked to advanced cancer features and studies only occasionally corrected the prognostic value for potential confounders (Jing et al. 2021a; Luo et al. 2017; Zhang et al. 2020; Lei et al. 2021; Sun et al. 2021). Studies investigating the effect of inhibition of PSMD14 show promising results in vitro and in vivo (Li et al. 2017; Jing et al. 2021a, b; Song et al. 2017; Yu et al. 2019).

Since a previous study has shown an association of PSMD14 mRNA and protein expression with advanced stages and prognosis (without correction for confounders), this study aimed to further elucidate whether PSMD14 is an independent prognostic marker in HNSCC. Therefore, PSMD14 copy number variation (CNV) and mRNA expression were evaluated in a primary cohort and protein expression in a second cohort. PSMD14 levels were investigated for their independent association with survival and correlation with clinicopathological data.

## Patients and methods

### The Cancer Genome Atlas dataset

Patient data were extracted from cBioportal.org from “The Cancer Genome Atlas (TCGA), Firehose Legacy” and supplemented with “TCGA PanCancer Atlas” (Liu et al. 2018) and “TCGA, Nature 2015” (Gatta et al. 2000s) as described before (Schnoell et al. 2021). Eleven patients were excluded due to missing or incomplete data and patients with an overall survival (OS) of less than two months due to possible postoperative complications. mRNA expression (RNA Seq V2 RSEM) was extracted from cBioportal.org on 16 April 2021 and stratified into low and high expression at a cutoff at a *z*-score > 0 for high expression. Copy number variations (CNV) of PSMD14 were extracted from UCSC Xena on 15 November 2021 (Goldman et al. 2020). Expression was divided into high and low expression at a cutoff at the median CNV.

### Tissue microarray dataset

A tissue microarray (TMA) containing 115 patients diagnosed with HNSCC was analyzed for the secondary cohort. Patients were included if they were diagnosed with HNSCC between 2002 and 2012 and received surgery and postoperative radio(chemo)therapy. Exclusion criteria were a second primary carcinoma, distant metastasis, prior cancer treatment, immunosuppression, or external treatment. The study was approved by the ethics committee of the Medical University of Vienna (EK1262/2019).

The TMA was established using a Galileo TMA CK Series—HTS Tissue computer assisted TMA Microarray Platform (Integrated Systems Engineering Srl, Milan, Italy). In short, three cylindrical cores (2 mm in diameter) per patient were taken from representative tumor areas from formalin-fixed, paraffin-embedded tissue. Histology was confirmed by hematoxylin–eosin staining.

### Immunohistochemistry

Immunohistochemistry of PSMD14 (HPA002114-25UL, Sigma Aldrich, St. Louis, MO, USA) was visualized using the Lab Vision Ultra kit (Thermo Scientific, Waltham, MA, USA) as described before. The antibody concentration and antibody retrieval buffer were assessed prior to TMA staining. Tissue slides were dewaxed and hydrated and the endogenous hydrogenase was blocked with H_2_O_2_. Subsequently, antibody retrieval was performed using EDTA buffer in a microwave. Next, Ultra V Block was applied followed by the incubation with PSMD14 1:500 for 1 h at room temperature. Afterward, the tissue slides were incubated with the primary antibody enhancer and horseradish peroxidase enhancer. Visualization of staining was performed using the UltraVision Plus Detection System DAB Plus Substrate System (Thermo Scientific, Fremont, CA, USA). Tissue was then counterstained with hematoxylin Gill III (Merck, Darmstadt, Germany) and scanned using an Olympus BH-2 microscope (Olympus, Tokyo, Japan). Staining intensity was analyzed using QuPath (Version 0.2.3) as described before (Schnoell et al. 2021). A score was formed to include the staining intensity and percentage of stained cells (3× % of cells with high positive staining + 2× % of cells with moderately positive staining + 1× % of cells with low positive staining). Protein expression was stratified using a score ≥ 30 as cutoff for high PSMD14 expression.

### Statistical analysis

Stata (Stata Corp., College Station, TX, USA) and Prism GraphPad software (GraphPad Software, Inc., La Jolla, CA, USA) were used to perform statistical analysis. Correlation between PSMD14 and clinicopathological data were assessed using Fisher’s exact test or Chi-squared test. Overall survival (OS) and disease-free survival (DFS) were calculated from the date of diagnosis to the date of death or recurrence, respectively. Survival was visualized with Kaplan Meier curves. Association with survival was analyzed using log-rank test and a cox-proportional hazard regression model.

## Results

### Primary dataset (TCGA)

Patient characteristics of the primary dataset are displayed in Table 1, left column. Five hundred and ten patients were included in the TCGA. Three hundred and seventy-eight patients (74%) were male and 307 (60%) had an oropharyngeal tumor. Seventy-six patients (15%) were HPV positive. The median age at diagnosis was 60.5 (53–68) years. The median observation period was 2.9 (1.8–4.6) years. The median OS was 4.7 (1.4–12.8) years and the median DFS was 4.7 (1.1–17.6) years. Most patients were treated with surgery and postoperative radiotherapy (*n* = 189, 37%).

**Table 1:** Baseline characteristics of the primary (TCGA) and secondary (TMA) HNSCC cohort

	Primary dataset (TCGA)		Secondary dataset (TMA)	
	Total (*n* = 510)	Percent (%)	Total (*n* = 115)	Percent (%)
Gender				
Female	132	26	27	23
Male	378	74	88	77
Age				
< 60	231	45	62	54
≥ 60	279	55	53	46
Primary				
Oral cavity	307	60	31	27
Oropharynx	79	15	52	45
Hypopharynx	10	2	21	18
Larynx	114	22	11	10
HPV				
Negative	413	81	88	77
Positive	76	15	25	22
*x*	21	4	2	2
T stage				
1	34	7	21	18
2	148	29	62	54
3	133	26	20	17
4	180	35	12	10
*x*	15	3	0	0
N stage				
0	237	46	24	21
1	81	16	29	25
2	162	32	62	54
3	9	2	0	0
*x*	21	4	0	0
M stage				
0	480	94	80	70
1	6	1	0	0
*x*	24	5	35	30
Staging				
I	20	4	2	2
II	94	18	16	14
III	103	20	29	25
IV	280	55	68	59
*x*	13	3	0	0
Smoker				
Never/ex	323	63	51	44
Active	173	34	64	56
*x*	14	3	0	0
Radiotherapy				
No	86	17	0	0
Adjuvant	189	37	115	100
Primary	18	4	0	0
Neoadjuvant	6	1	0	0
*x*	211	41	0	0
Pharmaceutical therapy				
No	129	25	96	83
Adjuvant	113	22	19	17
Primary	4	1	0	0
Neoadjuvant	8	2	0	0
*x*	256	50	0	0

### Association of PSMD14 mRNA expression and CNV with clinicopathological data

To assess the association of PSMD14 mRNA expression and CNV with clinicopathological features, mRNA expression was stratified into high and low expression and correlation analysis was performed using Fisher’s exact test or Chi-squared test.

mRNA expression of PSMD14 was high in 222 patients (44%). High expression of PSMD14 mRNA was associated with locally advanced stages (T3-4, *p* = 0.001), advanced overall staging (III-IV, *p* = 0.040) and an HPV negative status (*p* = 0.011). CNV was high in 252 patients (50%). High CNV was associated with locally advanced stages (T3-4, *p* = 0.044) and tobacco use (*p* = 0.014). PSMD14 mRNA expression moderately correlated with CNV (chi^2^ *p* < 0.001, *r* = 0.529).

### Analysis of PSM14 mRNA expression and CNV with prognosis

Next, PSMD14 mRNA expression and CNV were investigated for their association with OS and DFS using log-rank test and an uni- and multivariable cox-proportional hazard regression model.

Patients with high PSMD14 mRNA showed a shorter median OS (5.5 vs. 3.1 years, *p* = 0.013) and DFS (5.1 vs. 4.0 years, *p* = 0.032; Fig. 2). Patients with a high PSMD14 mRNA expression showed an increased risk for death (HR 1.41, 95% CI 1.07–1.85, *p* = 0.014) and recurrence (HR 1.36, 95% CI 1.02–1.80, *p* = 0.033) in univariable analysis (Table 2). Additional analysis of the continuous mRNA *z* scores revealed an increased risk for death (HR 1.19, 95% CI 1.07–1.32, *p* = 0.001) and recurrence (HR 1.16, 95% CI 1.04–1.29, *p* = 0.007). However, these results did not prevail in multivariable analysis after correction for stage, smoker and HPV status. CNV was not associated with OS or DFS (Fig. 2; Table 2).

**Table 2:** Univariable and multivariable analysis of overall survival (OS) and disease-free survival (DFS) and PSMD14 mRNA expression, copy number variation (CNV) or protein expression

	Univariable			Multivariable		
	HR	95% CI	*p* value	HR	95% CI	*p* value
**Overall survival**						
TCGA						
PSMD14 mRNA high vs. low	1.41	1.07–1.85	**0.014**	1.24	0.93–1.65	0.135
PSMD14 mRNA *z*-score	1.19	1.07–1.32	**0.001**	1.12	1.00–1.25	0.055
PSMD14 CNV high vs. low	1.09	0.83–1.43	0.539	0.94	0.71–1.26	0.685
PSMD14 CNV *z*-score	1.66	0.64–4.34	0.297	0.98	0.37–2.64	0.974
TMA						
PSMD14 protein high vs. low	1.66	0.96–2.86	0.070	1.39	0.96–2.86	0.259
PSMD14 protein score	1.00	1.00–1.01	0.467	1.00	1.00–1.01	0.957
**Disease-free survival**						
TCGA						
PSMD14 mRNA high vs. low	1.36	1.02–1.8	**0.033**	1.28	0.95–1.72	0.105
PSMD14 mRNA *z*-score	1.16	1.04–1.29	**0.007**	1.12	0.99–1.25	0.069
PSMD14 CNV high vs. low	1.04	0.79–1.38	0.771	1.00	0.74–1.35	0.999
PSMD14 CNV *z*-score	0.94	0.34–2.62	0.905	0.69	0.24–1.98	0.496
TMA						
PSMD14 protein high vs. low	1.25	0.66–2.37	0.500	1.07	0.55–2.06	0.845
PSMD14 protein score	1.01	1.00–1.02	0.263	1.00	0.99–1.01	0.542

A *p* value below 0.05 was considered significant (bold)

*HR* hazard ratio, *CI* confidence interval

### Secondary dataset (TMA)—analysis of PSMD14 protein expression

To validate the findings of the TCGA cohort, protein expression of PSMD14 was assessed using immunohistochemistry in a secondary cohort and associated with clinicopathological data and patient outcome.

**Fig. 1:**
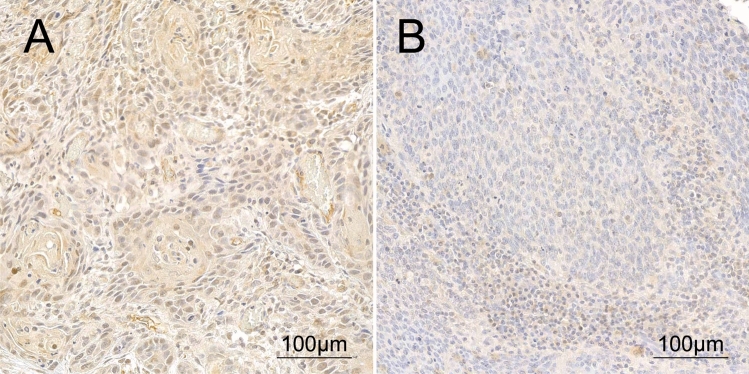
**A** High and **B** low protein expression of PSMD14 in HNSCC

One hundred fifteen patients were included in this dataset. Patient characteristics of the secondary dataset are described in Table 1, right column. The median age at diagnosis was 59 (53–63) years. The median observation period was 8.9 (6.3–11.8) years. The median OS was 9.9 (2.61-not reached) years and the median DFS was 13.1 (2.7-not reached) years. Protein expression was assessed using immunohistochemistry (Fig. 1). The median protein score was 33 (15–60). Patients were stratified at a protein expression score of 30. Sixty-four patients (56%) showed high expression and 51 patients (44%) showed low expression of PSMD14. Within the high and low expression groups, the median expression scores were 53 (38–78) and 12 (6–19), respectively. Eight patients (7%) were PSMD14 negative (expression score < 5).

**Fig. 2:**
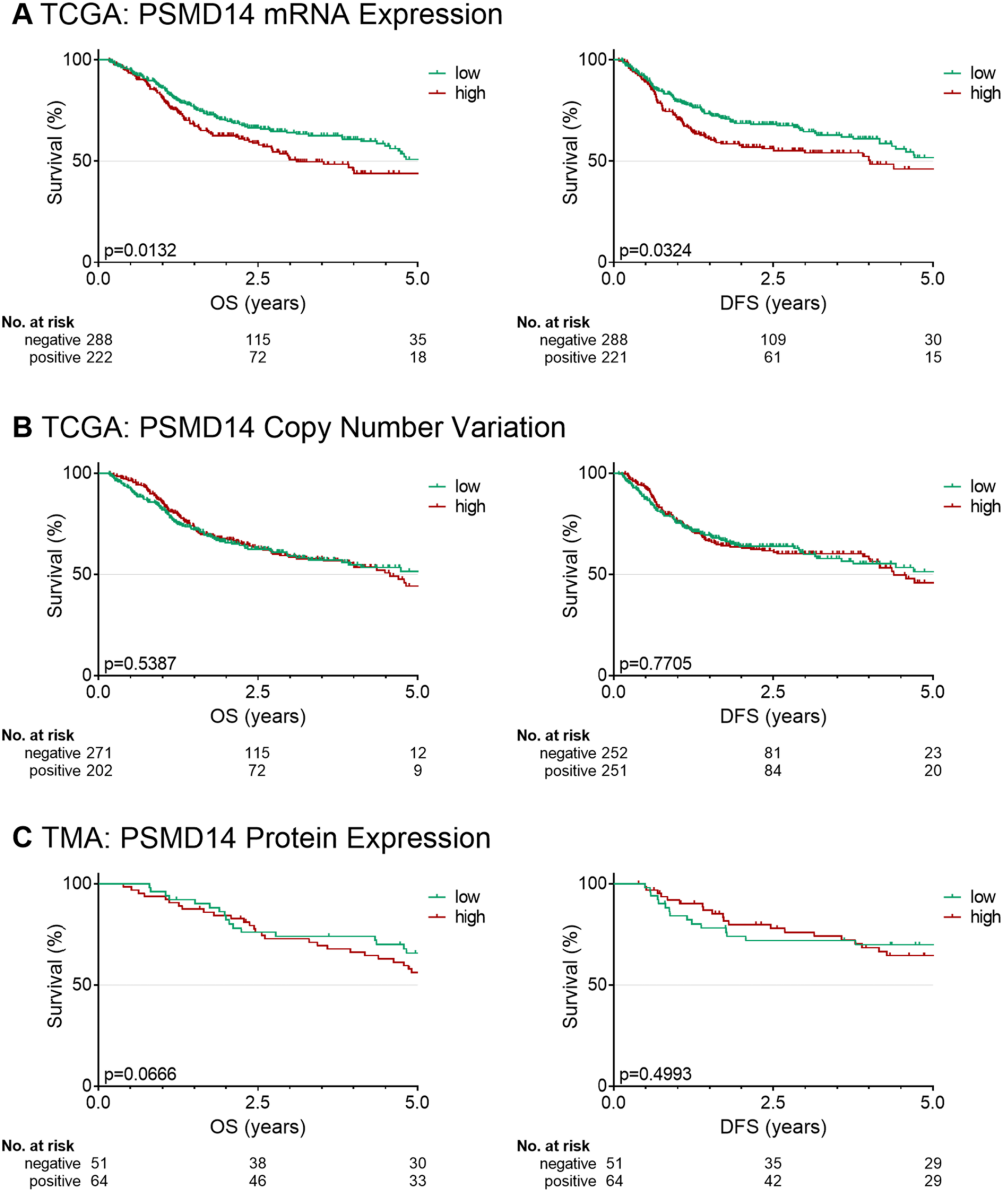
Kaplan–Meier survival curves of overall survival (OS) and disease-free survival (DFS) for PSMD14. **A** mRNA expression, **B** copy number variation (CNV), or **C** protein expression. *p*: log-rank *p* value

High PSMD14 protein expression was associated with lymph node metastasis (N1-3, *p* = 0.002) and advanced stages (III–IV, *p* = 0.002; Table 3). Protein expression was not associated with OS or DFS (Table 2, Fig. 2).

**Table 3:** Correlation analysis for associations between mRNA expression, copy number variation (CNV) and protein expression of PSMD14 and clinicopathological features

	Primary dataset (TCGA)			Primary dataset (TCGA)			Secondary dataset (TMA)		
	PSMD14 mRNA			PSMD14 CNV			PSMD14 protein		
	Low	High	*p* value	Low	High	*p* value	Low	High	*p* value
Sex									
Female	70 (24%)	62 (28%)		74 (29%)	56 (22%)		12 (24%)	15 (23%)	
Male	218 (76%)	160 (72%)	0.354	178 (71%)	196 (78%)	0.067	39 (76%)	49 (77%)	0.991
Age									
< 60	141 (49%)	90 (41%)		117 (46%)	112 (44%)		32 (63%)	30 (47%)	
≥ 60	147 (51%)	132 (59%)	0.058	135 (54%)	140 (56%)	0.655	19 (37%)	34 (53%)	0.090
T stage									
T1–2	120 (43%)	62 (29%)		100 (41%)	80 (32%)		38 (75%)	45 (70%)	
T3–4	159 (57%)	154 (71%)	**0.001**	143 (59%)	167 (68%)	**0.044**	13 (25%)	19 (30%)	0.618
N stage									
N0	131 (48%)	106 (50%)		123 (51%)	111 (46%)		18 (35%)	7 (11%)	
N1–3	144 (52%)	108 (50%)	0.677	118 (49%)	132 (54%)	0.238	33 (65%)	57 (89%)	**0.002**
Staging									
I–II	74 (26%)	40 (19%)		62 (25%)	50 (20%)		14 (27%)	4 (6%)	
III–IV	207 (74%)	176 (81%)	**0.040**	183 (75%)	197 (80%)	0.181	37 (73%)	60 (94%)	**0.002**
HPVhr									
Negative	223 (81%)	190 (89%)		205 (83%)	207 (86%)		40 (80%)	48 (76%)	
Positive	53 (19%)	23 (11%)	**0.011**	41 (17%)	35 (14%)	0.502	10 (20%)	15 (24%)	0.628
Smoker									
Never/ex	187 (67%)	136 (63%)		173 (71%)	147 (60%)		27 (53%)	24 (38%)	
Active	94 (33%)	79 (37%)	0.446	72 (29%)	98 (40%)	**0.014**	24 (47%)	40 (63%)	0.098

Analysis was performed using Fisher’s exact test or Chi-squared test. A *p* value below 0.05 was considered significant (bold)

*HPVhr* HPV high risk

## Discussion

HNSCC is the sixth most frequently diagnosed cancer worldwide. Treatment de-escalation is currently under investigation in HPV positive tumors because of the favorable prognosis (Mirghani and Blanchard 2018; Tawk et al. 2022). To date, there are no other biomarkers in clinical use to identify patients with a more aggressive phenotype. PSMD14 is an essential part of the proteasome and has been linked to prognosis in various cancers (Jing et al. 2021a; Lv et al. 2019; Wang et al. 2019; Zhu et al. 2018; Luo et al. 2017; Song et al. 2017; Zhang et al. 2020; Lei et al. 2021). In HNSCC, a first study has shown an association of PSMD14 mRNA and protein expression with advanced stages and prognosis, however, no correction for confounders was reported. Furthermore, inhibition of PSMD14 has shown promising results in vitro and in vivo (Jing et al. 2021a, b; Lv et al. 2019; Zhang et al. 2020). Therefore, the expression of PSMD14 mRNA, protein and CNV was investigated in two HNSCC cohorts and evaluated as an independent prognostic marker.

PSMD14 mRNA expression and CNV was high in 44 and 50% of patients, respectively, and protein expression of PSMD14 was high in 56%. High PSMD14 mRNA expression was associated with advanced T and N stage and a negative HPV status. In accordance, high CNV values were associated with a high T stage and active smokers. In the secondary dataset, high protein expression was associated with a positive N stage and advanced overall stage. These results are in line with literature. PSMD14 expression was high in 42% of HNSCC (Jing et al. 2021a), 66% of non-small cell lung cancer (Lei et al. 2021) and 64% of ovarian cancer (Sun et al. 2021). Furthermore, high PSMD14 levels were associated with advanced cancer stages in various cancer types (Jing et al. 2021a; Luo et al. 2017; Zhang et al. 2020; Lei et al. 2021; Sun et al. 2021). Since expression of PSMD14 is associated with tumor promotion through interaction with the transcription factor E2F1 (Jing et al. 2021a; Wang et al. 2015), our results further support that PSMD14 plays a role in cancer progression of HNSCC. A first preclinical study shows promising anticancer results of the PSMD14 inhibitor thiolutin in HNSCC (Jing et al. 2021a). Furthermore, inhibition of PSMD14 with o-phenanthroline, thiolutin and capzimin showed anticancer effects in other cancer types (Li et al. 2017; Jing et al. 2021b; Lv et al. 2019; Song et al. 2017). Based on our data, the evaluation of PSMD14 as a therapeutic target is recommended in HNSCC, especially in advanced stages, due to the more common upregulation.

Next, we analyzed the association of PSMD14 with prognosis. High PSMD14 mRNA expression was associated with a shorter median OS and DFS. Furthermore, high categorical and continuous mRNA expression was associated with a worse OS and DFS in univariable analysis. However, since high PSMD14 expression was associated with poor prognostic factors (advance stages, HPV negative status), we further performed multivariable analysis. After correction for possible confounders (including staging, HPV and smoker status), PSMD14 mRNA expression did not show an association with disease outcome. In contrast, when CNV and protein expression were analyzed, there was no association with OS or DFS. While Jing et al. concluded that high PSMD14 protein expression is a prognostic factor in HNSCC and is also associated with advanced stages in their study, no multivariable analysis was reported to correct for confounders (Jing et al. 2021a). Similarly, high expression of PSMD14 is associated with a worse prognosis in multiple myeloma, esophageal, ovarian, breast cancer and osteosarcoma. Although expression is associated with prognosis and occasionally with advanced cancer features in these studies, correction of the prognostic relevance of PSMD14 for potential confounders was not described (Lv et al. 2019; Luo et al. 2017; Song et al. 2017; Sun et al. 2021; Gong and Wei 2021). In accordance with our results, Zhang et al. revealed that PSMD14 is associated with OS and DFS in lung adenocarcinoma. However, after multivariable analysis, PSMD14 failed to show an independent association (Zhang et al. 2020). In contrast, PSMD14 is an independent prognostic factor in hepatocellular carcinoma and non-small cell lung cancer (Wang et al. 2019; Lei et al. 2021). Altogether, these results indicate that PSMD14 plays a role in advanced cancers. The question remains whether PSMD14 may serve as an independent prognostic biomarker in HNSCC or its association with prognosis is a result of upregulation during cancer progression.

There are limitations of this study that need to be discussed. First, mRNA and CNV expression were compared to protein expression in a second cohort. Although correlation of mRNA and protein at the bulk level is assumed, protein expression may be modified by post-transcriptional processes (Liu et al. 2016). Second, interpretation of the results is limited due to the retrospective design. Selection bias may apply; however, the risk was reduced by including a homogenously treated secondary cohort. Third, the stratification for high and low expression was determined using the median as an empiric cutoff. Thus, interpretation of the results in comparison to other studies is difficult as the cutoff may vary. Last, protein expression was analyzed in three TMA cores taken from representative tumor areas using QuPath. However, the distribution of protein expression within the tumor may vary.

In conclusion, the results of this retrospective study indicate that high PSMD14 is associated with advanced stages in HNSCC. Therefore, further evaluation of PSMD14 as a therapeutic target is warranted, especially in these subgroups. While PSMD14 mRNA expression was associated with prognosis in univariable Cox regression, this may result from the association with poor prognosticators.

## Data Availability

The datasets of this study are available from the corresponding author on reasonable request.

## Abbreviations

CI: confidence interval
CNV: copy number variations
DFS: Disease-free survival
HR: hazard ratio
OS: overall survival
PSMD14: 26S proteasome non-ATPase regulatory subunit 14
TCGA: The Cancer Genome Atlas
TMA: tissue microarray

## References

[CR1] Gatta G, Botta L, Sánchez MJ (2015). Prognoses and improvement for head and neck cancers diagnosed in Europe in early 2000s: the EUROCARE-5 population-based study. Eur J Cancer.

[CR2] Goldman MJ, Craft B, Hastie M (2020). Visualizing and interpreting cancer genomics data via the Xena platform. Nat Biotechnol.

[CR3] Gong Y, Wei Z (2021) Identification of PSMD14 as a potential novel prognosis biomarker and therapeutic target for osteosarcoma. Cancer Rep e1522 10.1002/cnr2.1522 PMC9327663 34383385

[CR4] Jing C, Duan Y, Zhou M (2021). Blockade of deubiquitinating enzyme PSMD14 overcomes chemoresistance in head and neck squamous cell carcinoma by antagonizing E2F1/Akt/SOX2-mediated stemness. Theranostics.

[CR5] Jing C, Li X, Zhou M (2021). The PSMD14 inhibitor Thiolutin as a novel therapeutic approach for esophageal squamous cell carcinoma through facilitating SNAIL degradation. Theranostics.

[CR6] Johnson DE, Burtness B, Leemans CR, Lui VWY, Bauman JE, Grandis JR (2020). Head and neck squamous cell carcinoma. Nat Rev Dis Prim.

[CR7] Lei J, Liu X, Liu W, Zhang Y, Liu Z (2021). The prognostic value of USP14 and PSMD14 expression in non- small cell lung cancer. Ann Transl Med.

[CR8] Li J, Yakushi T, Parlati F (2017). Capzimin is a potent and specific inhibitor of proteasome isopeptidase Rpn11. Nat Chem Biol.

[CR9] Liu Y, Beyer A, Aebersold R (2016). On the dependency of cellular protein levels on mRNA abundance. Cell.

[CR10] Liu J, Lichtenberg T, Hoadley KA (2018). An integrated TCGA pan-cancer clinical data resource to drive high-quality survival outcome analytics. Cell.

[CR11] Luo G, Hu N, Xia X, Zhou J, Ye C (2017). RPN11 deubiquitinase promotes proliferation and migration of breast cancer cells. Mol Med Rep.

[CR12] Lv J, Zhang S, Wu H (2020). Deubiquitinase PSMD14 enhances hepatocellular carcinoma growth and metastasis by stabilizing GRB2. Cancer Lett.

[CR13] Mirghani H, Blanchard P (2018). Treatment de-escalation for HPV-driven oropharyngeal cancer: where do we stand?. Clin Transl Radiat Oncol.

[CR14] Pulte D, Brenner H (2010). Changes in survival in head and neck cancers in the late 20th and early 21st century: a period analysis. Oncologist.

[CR15] Schnoell J, Jank BJ, Kadletz-Wanke L (2021). Transcription factors CP2 and YY1 as prognostic markers in head and neck squamous cell carcinoma: analysis of The Cancer Genome Atlas and a second independent cohort. J Cancer Res Clin Oncol.

[CR16] Shin JY, Muniyappan S, Tran N-N, Park H, Lee SB, Lee B-H (2020). Deubiquitination reactions on the proteasome for proteasome versatility. Int J Mol Sci.

[CR17] Song Y, Li S, Ray A (2017). Blockade of deubiquitylating enzyme Rpn11 triggers apoptosis in multiple myeloma cells and overcomes bortezomib resistance. Oncogene.

[CR18] Sun T, Liu Z, Bi F, Yang Q (2021). Deubiquitinase PSMD14 promotes ovarian cancer progression by decreasing enzymatic activity of PKM2. Mol Oncol.

[CR19] Tawk B, Debus J, Abdollahi A (2022). Evolution of a paradigm switch in diagnosis and treatment of HPV-driven head and neck cancer—striking the balance between toxicity and cure. Front Pharmacol.

[CR20] Wang B, Ma A, Zhang L (2015). POH1 deubiquitylates and stabilizes E2F1 to promote tumour formation. Nat Commun.

[CR21] Wang B, Xu X, Yang Z (2019). POH1 contributes to hyperactivation of TGF-β signaling and facilitates hepatocellular carcinoma metastasis through deubiquitinating TGF-β receptors and caveolin-1. EBioMedicine.

[CR22] Yu W, Li J, Wang Q (2019). Targeting POH1 inhibits prostate cancer cell growth and enhances the suppressive efficacy of androgen deprivation and docetaxel. Prostate.

[CR23] Zhang L, Xu H, Ma C (2020). Upregulation of deubiquitinase PSMD14 in lung adenocarcinoma (LUAD) and its prognostic significance. J Cancer.

[CR24] Zhu R, Liu Y, Zhou H (2018). Deubiquitinating enzyme PSMD14 promotes tumor metastasis through stabilizing SNAIL in human esophageal squamous cell carcinoma. Cancer Lett.

